# Genotyping Hepatitis B virus by Next-Generation Sequencing: Detection of Mixed Infections and Analysis of Sequence Conservation

**DOI:** 10.3390/ijms25105481

**Published:** 2024-05-17

**Authors:** Eva Dopico, Marta Vila, David Tabernero, Josep Gregori, Ariadna Rando-Segura, Beatriz Pacín-Ruíz, Laura Guerrero, Itziar Ubillos, Miguel J. Martínez, Josep Costa, Josep Quer, Javier Pérez-Garreta, Alejandra González-Sánchez, Andrés Antón, Tomás Pumarola, Mar Riveiro-Barciela, Roser Ferrer-Costa, Maria Buti, Francisco Rodríguez-Frías, Maria Francesca Cortese

**Affiliations:** 1 Department of Microbiology, Metropolitana Sud Territorial Clinical Laboratory, Bellvitge University Hospital, Institut Català de la Salut (ICS), 08907 Hospitalet de Llobregat, Spain; evadopico@gmail.com (E.D.); laura.guerrero.latorre@gmail.com (L.G.); itziar.ubillos@gmail.com (I.U.); 2 Bellvitge Biomedical Research Institute (IDIBELL), 08908 Hospitalet de Llobregat, Spain; 3 Liver Unit, Microbiology Department, Vall d’Hebron Institut de Recerca (VHIR), Vall d’Hebron Hospital Universitari, Vall d’Hebron Barcelona Hospital Campus, 08035 Barcelona, Spain; marta.vila.salvador@vhir.org (M.V.); ariadna.rando@vallhebron.cat (A.R.-S.); beatriz.pacin@vhir.org (B.P.-R.); javierperezgarreta@gmail.com (J.P.-G.); maria.cortese@vhir.org (M.F.C.); 4 Centro de Investigación Biomédica en Red de Enfermedades Hepáticas y Digestivas (CIBERehd), Instituto de Salud Carlos III, 28029 Madrid, Spain; josep.quer@vhir.org (J.Q.); mar.riveiro@vallhebron.cat (M.R.-B.); mariaasuncion.buti@vallhebron.cat (M.B.); frarodri@gmail.com (F.R.-F.); 5 Liver Diseases-Viral Hepatitis, Liver Unit, Vall d’Hebron Institut de Recerca (VHIR), Vall d’Hebron Hospital Universitari, Vall d’Hebron Barcelona Hospital Campus, 08035 Barcelona, Spain; josep.gregori@gmail.com; 6 Virology Section, Microbiology Department, Vall d’Hebron Hospital Universitari, Vall d’Hebron Barcelona Hospital Campus, 08035 Barcelona, Spain; alejandra.gonzalez@vallhebron.cat (A.G.-S.); andres.anton@vallhebron.cat (A.A.); tomas.pumarola@vallhebron.cat (T.P.); 7 Department of Microbiology, Hospital Clínic, Universitat de Barcelona, 08036 Barcelona, Spain; myoldi@clinic.cat (M.J.M.); jcostacamps1955@gmail.com (J.C.); 8 ISGlobal, Hospital Clínic, Universitat de Barcelona, 08036 Barcelona, Spain; 9 Centro de Investigación Biomédica en Red de Enfermedades Infecciosas (CIBERINFEC), Instituto de Salud Carlos III, 28029 Madrid, Spain; 10 Biochemistry and Molecular Biology Department, Universitat Autònoma de Barcelona (UAB), 08193 Bellaterra, Spain; 11 Respiratory Virus Unit, Microbiology Department, Vall d’Hebron Institut de Recerca (VHIR), Vall d’Hebron Hospital Universitari, Vall d’Hebron Barcelona Hospital Campus, 08035 Barcelona, Spain; 12 Liver Unit, Internal Medicine Department, Vall d’Hebron Hospital Universitari, Vall d’Hebron Barcelona Hospital Campus, 08035 Barcelona, Spain; 13 Clinical Biochemistry, Drug Delivery and Therapy (CB-DDT) Research Group, Vall d’Hebron Institut de Recerca (VHIR), Vall d’Hebron Hospital Universitari, Vall d’Hebron Barcelona Hospital Campus, 08035 Barcelona, Spain; roser.ferrer@vallhebron.cat; 14 Biochemistry Department, Vall d’Hebron Hospital Universitari, Vall d’Hebron Barcelona Hospital Campus, 08035 Barcelona, Spain; 15 Department of Basic Sciences, Universitat Internacional de Catalunya, 08017 Barcelona, Spain

**Keywords:** hepatitis B virus, genotypes, next-generation sequencing, quasispecies, conservation, information content, Distance-Based discrimination method, preS, hepatitis B X gene, RNA interference

## Abstract

Our aim was to develop an accurate, highly sensitive method for HBV genotype determination and detection of genotype mixtures. We examined the preS and 5′ end of the HBV X gene (5X) regions of the HBV genome using next-generation sequencing (NGS). The 1852 haplotypes obtained were subjected to genotyping via the Distance-Based discrimination method (DB Rule) using two sets of 95 reference sequences of genotypes A–H. In clinical samples from 125 patients, the main genotypes were A, D, F and H in Caucasian, B and C in Asian and A and E in Sub-Saharan patients. Genotype mixtures were identified in 28 (22.40%) cases, and potential intergenotypic recombination was observed in 29 (23.20%) cases. Furthermore, we evaluated sequence conservation among haplotypes classified into genotypes A, C, D, and E by computing the information content. The preS haplotypes exhibited limited shared conserved regions, whereas the 5X haplotypes revealed two groups of conserved regions across the genotypes assessed. In conclusion, we developed an NGS-based HBV genotyping method utilizing the DB Rule for genotype classification. We identified two regions conserved across different genotypes at 5X, offering promising targets for RNA interference-based antiviral therapies.

## 1. Introduction

Hepatitis B virus (HBV) causes a world-wide prevalent infection, affecting an estimated 296 million people with chronic HBV infection as of 2019 [1]. Moreover, it stands as the main cause of hepatocellular carcinoma (HCC) due to viral infection [2]. Despite the availability of antiviral therapeutic protocols, which effectively suppress viral replication, the cure for infection remains elusive since it is currently not possible to eradicate the intracellular covalently closed circular DNA (cccDNA) and the HBV sequences integrated into the host genome [3].

Notably, the cccDNA forms a histone-associated mini-chromosome that serves as the template for transcription of all viral messenger RNAs, including the pregenomic RNA. This RNA is subsequently encapsidated and reverse-transcribed within core particles to form new molecules of relaxed circular DNA [4]. This last step is performed by the error-prone HBV polymerase, which introduces approximately 10^−5^ nucleotide (nt) substitutions/site/year [5]. This high nt substitution rate together with a high production of viral particles, estimated at around 10^11^ virions/day [6], results in highly heterogeneous viral populations comprising genetically closely related, though not identical, genomes forming a viral quasispecies [7].

Due to this high genetic heterogeneity, HBV genomes can be classified into phylogenetically related groups termed genotypes, defined by a divergence of more than 7.5% in their whole nt sequence. Furthermore, several sub-genotypes have been described, exhibiting nt sequence divergences ranging from 4% to 7.5% [8]. Apart from the genotype D, which is distributed worldwide (with a higher prevalence in North Africa, Europe, the Middle East, Central Asia and India), other genotypes presented distinct geographic distributions: genotype A predominates in Africa, Europe, and North America; genotypes B and C in the Asia–Pacific region; genotype E in West and Central Africa; genotypes F and H in Central America, South America (mainly F) and North America (mainly H); and genotype G in European and North American countries. Finally, genotype I has primarily been isolated in Southeast Asia, while genotype J has been observed in only one patient from Japan [9]. However, the current global people flow, particularly from high HBV endemicity regions to areas with low seroprevalence, such as Europe and North America, are significantly influencing this distribution [8].

From a clinical perspective, distinct genotypes have been associated with different outcomes of antiviral treatment and liver disease progression. For instance, in a previous report [10], patients infected with genotype B showed a better response to antiviral treatment with interferon-α (IFN-α) than those infected with genotypes C and D. Moreover, patients in whom more than one genotype was identified (i.e., genotype mixtures) exhibited the least sensitive response to IFN-α. Another study [11] showed that patients infected with genotype D had a significantly higher risk of developing HCC than those infected with genotype A, with a six-fold increased risk observed for individuals harboring A+D mixed genotypes. These findings reinforce the importance of determining HBV genotypes and their mixtures as potential predictors of liver disease progression in chronic hepatitis B (CHB) patients.

In the current landscape, various HBV genotyping methods exist, including reverse hybridization with Inno-LiPA, reverse dot blot assays, oligonucleotide microarrays, real-time PCR assays, etc. These methods offer high sensitivity in detecting known genotypes and their mixtures; however, their suitability may be affected by mutations within the HBV genome. For this reason, sequencing of the whole HBV genome followed by phylogenetic analysis is currently regarded as the “gold standard” approach for HBV genotyping [8,12]. Notably, the analysis of specific segments of the HBV genome as short as 300 nt has proven sufficient for accurate HBV genotype discrimination [13]. In this regard, sequencing amplicons encompassing genomic regions capable of effectively distinguishing viral genotypes using next-generation sequencing (NGS) may be an optimal strategy for determining HBV genotypes and detecting their mixtures with high sensitivity [14,15,16].

The aim of this study was to develop an accurate method for determining HBV genotypes and detecting their mixtures with high sensitivity in clinical samples. This method relies on NGS of two amplicons capable of distinguishing between different viral genotypes. The NGS raw data underwent processing through a bioinformatics pipeline, ultimately determining the genotype via genetic distance discrimination. Of note, this method enables high-throughput analysis of HBV quasispecies, which allowed us to utilize previously generated NGS data to explore quasispecies sequence conservation and compare them across different HBV genotypes.

## 2. Results

### 2.1. Hepatitis B Virus Genomic Regions Analyzed and Genotype Determination Using the Distance-Based Discrimination Method

In the present study, we used two amplicons for determining HBV genotypes, which had previously undergone successful NGS analysis in studies conducted by our group [17,18,19]. The first amplicon encompassed a region spanning nt positions 2837/2838/2844/2874 (genotypes F/B, C, D, E and H/A/G, respectively) to position 56, covering the entire preS1 and the 5′ end of the preS2 coding regions of the S gene, hereinafter referred to as preS. The second amplicon covered the hepatitis B X gene (*HBX*) 5′ end, spanning nt positions 1255 to 1611, hereinafter referred to as 5X. Moreover, the P gene overlapped with the S and *HBX* genes in the sequence of these amplicons. Regarding preS, while its 5′ end encoded the last four C-terminal end amino acids (aa) of the terminal peptide, most of its sequence codified the N-terminal region of the spacer domain. As for 5X, its sequence was included in the RNAse H domain coding sequence.

**Figure 1:**
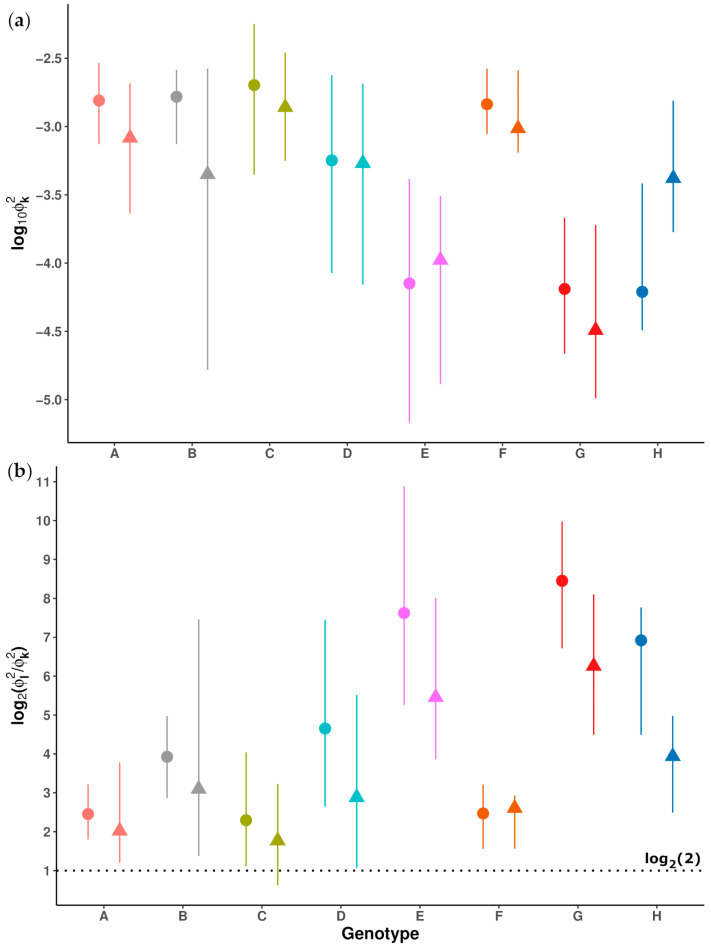
Summaries of the proximity function values from the reference sequences to their respective genotypes. Median values correspond to circles (preS) and triangles (5X), while minimal and maximal values are indicated by the lowest and the highest ends of lines crossing circles and triangles, respectively: (**a**) Base-10 logarithm of minimal, median and maximal lowest proximity functions values ($\Phi_{k}^{2}$); (**b**) Base-2 logarithm of minimal, median and maximal ratios between the two lowest proximity function values ($\Phi_{l}^{2}/\Phi_{k}^{2}$). The safety threshold of 2 for classifying a sequence into a given genotype is denoted by a dotted black line in the graph.

Each sequence obtained from preS and 5X was classified into an HBV genotype using the Distance-Based discrimination method (DB Rule) [20]. Briefly, the DB rule was used to define a proximity function ($\Phi_{t}^{2}$), which quantifies the genetic distance of each sequence to the *t*-th HBV genotype (A–H), taking into account the intraclass variability within each genotype. The calculation of this proximity function for a sequence to each genotype relies on a set of patterns (reference sequences) for each of them. In this study, we used two sets of 95 reference sequences, for preS and 5X, representing HBV genotypes A (11); B (19); C (21); D (17); E (7); F (10); G (5) and H (5) (Figure S1), obtained from a large set of HBV full genome sequences with assigned genotypes downloaded from the GenBank database (National Center for Biotechnology Information, USA). Each HBV sequence to be classified into a genotype (i.e., the problem sequence) was assigned to the genotype with the lowest $\Phi_{t}^{2}$ value, $\Phi_{k}^{2}=min\left(\Phi_{t}^{2}\right)$, corresponding to the *k*-th genotype (i.e., the genotype to which the haplotype showed the lowest genetic distance). A summary of $\Phi_{k}^{2}$ values between reference sequences and their respective genotypes, illustrating the intraclass variability of each genotype, is shown in Figure 1a.

Any computed $\Phi_{t}^{2}$ resulting in a negative value was taken as 0. The ratio between the two lowest $\Phi_{t}^{2}$ values ($\Phi_{l}^{2}/\Phi_{k}^{2}\geq 1$) was computed for each reference sequence (Figure S2) and for each problem sequence obtained from a patient sample, serving as a measure of classification confidence. A higher value of this ratio indicates greater confidence in the classification into the assigned genotype, indicating a less ambiguous result. Analysis of the $\Phi_{l}^{2}/\Phi_{k}^{2}$ values of the reference sequences to their respective genotypes showed that a ratio above 2 provides a reliable classification (Figure 1b), and this ratio was taken as the classification confidence score.

### 2.2. Analysis of Hepatitis B Virus Genotypes

#### 2.2.1. Next-Generation Sequencing Yield and Haplotype Genotyping

In the present study, we included 144 leftover serum or plasma clinical samples referred to the *Laboratoris Clínics Vall d’Hebron* (LCVH) or the *Laboratori Clínic Territorial Metropolitana Sud* (LCTMS) of the *Institut Català de la Salut* (Barcelona, Spain), with a median HBV-DNA level of 5.74 (interquartile range [IQR] 4.44–7.87) logIU/mL. Among these samples, 80 (55.56%) tested positive for hepatitis B e antigen (HBeAg). The samples were obtained from 129 CHB patients, of whom 71 (55.04%) were HBeAg positive at the time of inclusion in this study. Among them, 11 patients had at least 2 follow-up samples (a total of 26/144 samples; 18.06%). The HBeAg status of these 11 patients remained stable in all their follow-up samples.

The HBV-DNA was isolated from all samples and analyzed using NGS in both preS and 5X amplicons. After bioinformatics filtering, a total of 5,515,758 sequence reads were obtained, with 2,444,705 reads for preS and 3,071,053 reads for 5X. The reads with identical sequences were collapsed into 1852 haplotypes (i.e., unique sequences covering the full amplicon, with their corresponding frequencies in each quasispecies): 1030 from preS and 822 from 5X. The HBV genotype for each haplotype was determined using the DB Rule (Table 1).

**Figure 2:**
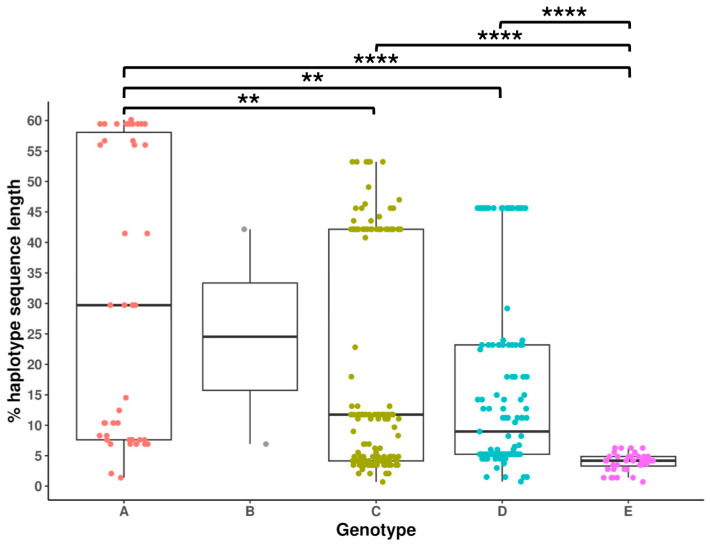
Boxplots representing the distribution of the percentages of the length of preS haplotype sequences affected by gaps, according to their hepatitis B virus genotype. Only the haplotypes from a baseline sample from each patient were included within these boxplots. The statistically significant *p*-values are obtained after applying a multiple pairwise comparison test (post-hoc Dunn test with Bonferroni correction) and are reported as asterisks: ** *p* < 0.01 and **** *p* < 0.0001.

Of note, 393 (38.16%) of the 1030 preS haplotypes showed gap/s (i.e., absence of at least 1 nt at a given position in a multiple alignment of their sequences, due to deletions (Del) or insertions (Ins)), while no gaps were observed in 5X haplotypes (Table 1). Among haplotypes with gaps, nearly all exclusively showed Del; only two genotype D haplotypes displayed Ins, one between positions 2855 and 2866, and the other between positions 31 and 34, combined with a larger Del from position 35 to the end of the analyzed sequence. The distribution of the percentages of the length of preS haplotype sequences affected by gaps in multiple alignments of different genotypes was significantly different between them (*p* < 0.01). As reported in Figure 2, the distribution of the percentages of haplotype sequence length affected by gaps significantly differed between genotype A and E haplotypes. Moreover, the distributions of these genotypes were also significantly different to those of genotypes C and D. Despite that the presence of gaps may increase classification ambiguity, none of the 393 preS haplotypes displaying gaps exhibited ($\Phi_{l}^{2}/\Phi_{k}^{2}$) score values below 2. This was true even for the genotype A, C and D haplotypes with more than 40% of their sequences deleted. Therefore, the classification of these haplotypes in their respective genotypes was considered safe.

#### 2.2.2. Analysis of Main Hepatitis B Virus Genotypes and Genotype Mixtures in Chronic Hepatitis B Patients

Since two genomic regions were utilized in this study, the assigned genotypes for each sample were compared between the two amplicons. Consequently, four samples from four different patients were excluded, as no paired reads from preS and 5X were obtained. Therefore, genotyping was performed on 140 serum or plasma clinical samples (median HBV-DNA levels 5.78 (4.46–7.90) logIU/mL), obtained from 125 CHB patients. Most of these patients were male (71, 56.80%), with a median age of 33 (27–44) years. Only 3 (2.40%) showed serological evidence of co-infection with hepatitis D virus (HDV) and 3 (2.40%) with human immunodeficiency virus (HIV). Detailed data for genotyping of these samples are provided in Table S1.

**Figure 3:**
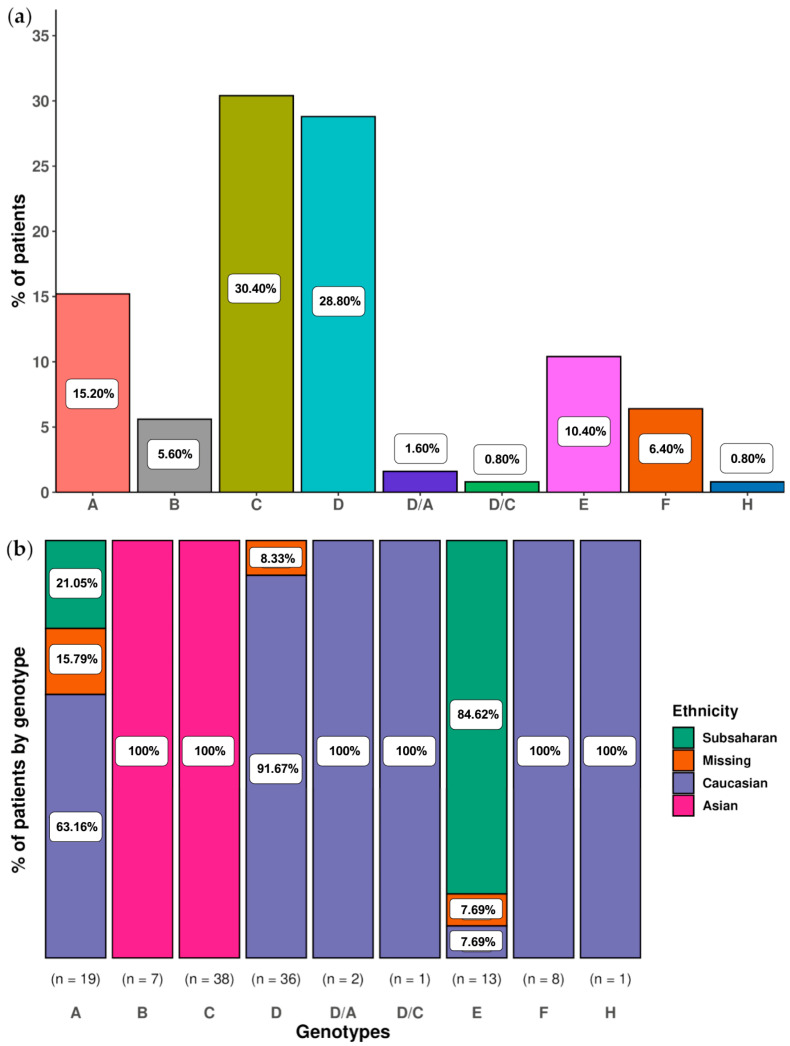
Main HBV genotype in the 125 patients analyzed: (**a**) Percentage of patients per main genotype; (**b**) Distribution of ethnicities among patients with the same main genotype. Data on ethnicity was not available for 7 patients (reported in orange).

In most of the 140 samples, the main genotype, determined by the highest sum of haplotype frequencies, matched in both amplicons. Notably, diverse HBV genotypes were represented among the main ones: D (31.43%), C (28.57%), A (15.71%), E (9.29%), with B and F at 5.71% each, and H at 1.43%. Only in 3 (2.14%) samples was the main genotype different in both amplicons. Among the 11 patients with at least 2 follow-up samples, the main genotype remained unchanged during the follow-up of the present study. To simplify the presentation of results, only their baseline sample was considered while assessing the distribution of the main genotypes and genotype mixtures in our CHB patient population. The distribution of the main genotypes in the 125 samples examined is displayed in Figure 3a. This distribution was influenced by the HBeAg status of patients (71 HBeAg positive and 53 HBeAg negative, with 1 sample where HBeAg status was unknown). Regarding the most prevalent main genotypes, genotypes A and D were mainly observed in HBeAg negative samples (5.63% HBeAg positive vs. 28.30% HBeAg negative, *p* < 0.01, and 21.13% of HBeAg positive vs. 39.62% of HBeAg negative, *p* = 0.02, respectively), while genotype C predominated in HBeAg positive samples (47.89% of HBeAg positive vs. 5.66% of HBeAg negative, *p* < 0.01). Furthermore, we explored the correlation between main genotypes and patient ethnicities (Figure 3b). The most widely represented ethnic group comprised Caucasians (58 patients, 46.40%) from different geographic origins including Spain, Eastern Europe, the Indian subcontinent, South America, etc. As expected, they exhibited diverse HBV genotypes, such as A, D, F, and H. Of note, in one Caucasian patient, genotype E emerged as the main genotype (patient 118, Table S1). This genotype was detected after viral replication reactivation post-liver transplantation. Additionally, in two Caucasian patients, genotype D was the main one in preS and genotype A in 5X (patients 6 and 125), while another showed genotype D in preS and genotype C in 5X (patient 98) (Table S1). All three patients displayed HBeAg negativity. The second most prevalent ethnicity was Asian (45 patients, 36%). Most Asian patients displayed C as the main genotype, thus contributing to its high prevalence among our cohort (Figure 3a), along with genotype B to a lesser extent. Lastly, Sub-Saharan patients (15, 12%) showed A and E as main genotypes.

Among the 125 samples examined, the main genotype was detected exclusively in both amplicons in 97 (77.60%) samples, while in 28 (22.40%) genotype mixtures were observed (Table 2), without statistically significant differences between proportions of samples showing genotype mixtures in preS (10/125, 8%) and 5X (18/125, 14.40%). Of these 28 samples, 17 were from Caucasian patients (29.31% of 58 samples obtained from this ethnic group). Notably, most of these samples (11) showed minor haplotypes classified into genotype C in either preS or 5X. Regarding the other ethnicities, genotype mixtures were detected in 5/45 (11.11%) samples from Asian and in 3/15 (20%) samples from Sub-Saharan patients. Minor genotype D haplotypes were identified in 4 out of 5 samples from Asian patients and in all 3 samples from Sub-Saharan patients. Of note, the genotype composition differed between the two amplicons in all samples with genotype mixtures (Table 2). Additionally, in patient 98, a distinct main genotype was identified in preS and 5X, despite the absence of genotype mixtures. Considering all the results together, variants potentially resulting from recombination between different genotypes (intergenotypic recombination) were observed in 29/125 (23.20%) samples.

Finally, we aimed to compare the results obtained using our high-throughput NGS-based method with Sanger sequencing, another sequencing method commonly used to determine the HBV genotype [21,22,23]. This comparative analysis was conducted on a group of 66 samples, selected depending on their availability for additional Sanger sequencing after NGS analysis. Notably, Sanger sequencing cannot detect nt polymorphisms comprising less than 20% of the quasispecies population [24], limiting its applicability in determining genotype mixtures. In this sense, the genotype obtained in each amplicon through Sanger sequencing (using the DB Rule in the same manner as for NGS haplotypes) was compared to the main genotype identified by NGS. Both methods showed complete agreement in the results obtained, confirming even the discrepancies between the main genotype in preS (D) and 5X (A) observed in patient 6 (Table S1).

### 2.3. Study of Sequence Conservation in the preS and 5X Amplicons across Different Genotypes

To study quasispecies sequence conservation in different HBV genotypes, we calculated the information content (IC) of each position in the haplotypes previously genotyped. The IC measures the degree of uncertainty in finding a specific nt at a given position in a multiple alignment of haplotypes, ranging from 0 (equal probability of finding any of the 4 nt in any haplotype) to 2 (100% probability of finding a specific nt in any haplotype, i.e., fully conserved position). This analysis was performed by genotype; thus, a multiple alignment was made for each set of haplotypes classified into the same genotype. However, we did not obtain enough haplotypes from genotypes B, F, and H to reliably identify the most conserved segments (i.e., windows) in haplotypes classified into these genotypes, and they were excluded from this analysis. In the remaining genotypes (A, C, D and E), we aimed to identify the most conserved regions shared between them in both amplicons.

In preS, the presence of gaps hindered the analysis of conservation and its comparison between different genotypes. Notably, in the alignments of genotypes A, C, D and E, we observed specific intragenotypic patterns of frequencies and distribution of gaps throughout the preS sequence (Figure S3), which may bias the comparison of sequence conservation between them. To limit these potential biases, we decided to focus the analysis of conservation on a short (144 nt in length) region limitedly affected by Ins and Del. This portion of the preS corresponded to the following genotype-specific positions: 2887–3030 for genotype A; 2881–3024 for genotype C; 2848–2991 for genotype D; and 2878–3021 for genotype E. These positions included the region encoding the 47 aa N-terminal domain of the HBV large surface protein (LHBs), involved in the attachment of the virus to the sodium-taurocholate co-transporting polypeptide (NTCP) [25]. Conversely, since the 5X did not show gaps in any haplotype, the whole haplotype sequences from genotypes A, C, D and E were included in the conservation study.

In the IC calculation at each position of both amplicons, we considered the frequencies of the different haplotypes to take into account their relative fitness while studying sequence conservation. After calculating the IC at each position, we performed a sliding window (SW) analysis, consisting of calculation of the mean IC in windows comprising 12-nt positions, moving forward with a 1-nt step between them. The 144-nt preS fragment and the 5X sequences showed genotype-specific IC profiles (Figures S4 and S5). To compare the sequence conservation across different viral genotypes, we first identified the most conserved windows in each of them. For this purpose, windows with mean IC values equal to or higher than the quantile 95 of all the windows obtained from the same amplicon and genotype were considered as the most conserved. Among these windows, those that were adjacent were concatenated, forming highly conserved regions. Finally, these regions were compared across different genotypes to identify pan-genotypic conserved regions.

**Figure 4:**
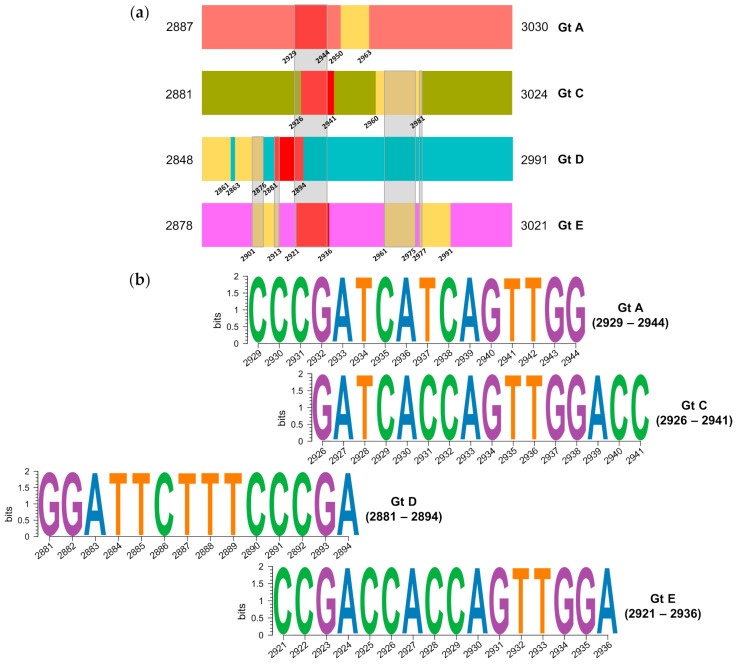
Analysis of conservation in the 144-nucleotide preS fragment: (**a**) Localization of the most conserved regions, with the genotype-specific positions of each of them. Regions with overlapping positions across all genotypes (A, C, D, and E) are shown in red, and portions shared between two or more regions in different genotypes are framed in grey; (**b**) Sequence logos of the conserved regions per genotype. Gt, Genotype.

Eleven highly conserved regions were observed for preS (Figure 4a). Among them, those between positions 2929 and 2944 (genotype A), 2926–2941 (genotype C), and 2921–2936 (genotype E) exhibited a high degree of sequence overlap, but only a small overlap with the region between positions 2881 and 2894 in genotype D (Figure 4b). Of note, these regions displayed a low incidence of gaps. In the conserved regions of genotypes A and C, each position included showed gaps in 2.23% and 6.08% of haplotype sequences assessed, respectively. In genotype D, only one haplotype contained a 12-nt Ins between positions 2855 and 2866, and no gaps were detected in genotype E haplotypes. Therefore, there were no large, conserved regions sharing the same sequence in all four genotypes.

**Figure 5:**
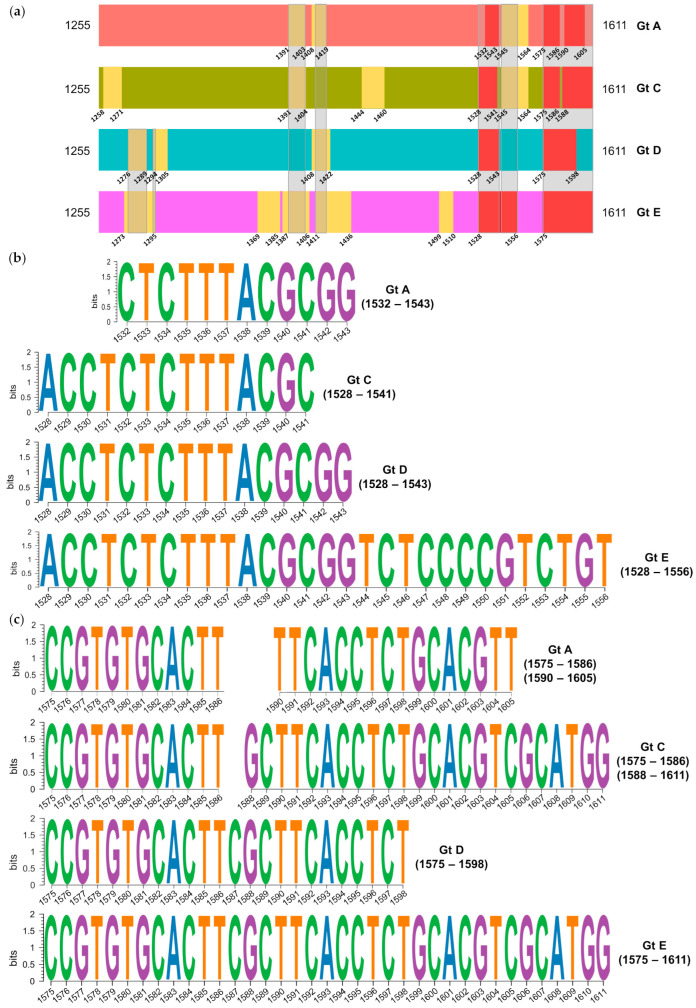
Analysis of conservation in the sequence of the 5X amplicon: (**a**) Localization of the most conserved regions, with the positions of each of them. Regions with overlapping positions across all genotypes (A, C, D, and E) are depicted in red, and the portions shared between two or more regions in different genotypes are framed in grey; (**b**) Sequence logos of the first group of pan-genotypic conserved regions per genotype; (**c**) Sequence logos of the second group of pan-genotypic conserved regions per genotype. Gt, Genotype.

In the 5X amplicon, 25 conserved regions were identified, 10 of which were grouped into two pan-genotypic conserved regions at the 3′ end of the amplicon (Figure 5a). The first group included the regions between positions 1532–1543 (genotype A), 1528–1541 (genotype C), 1528–1543 (genotype D), and 1528–1556 (genotype E). The second group comprised the regions between positions 1575 and 1586, and 1590–1605 (genotype A), 1575–1586, and 1588–1611 (genotype C), 1575–1598 (genotype D), and 1575–1611 (genotype E). Both groups of pan-genotypic conserved regions showed a high degree of intergenotype sequence similarity (Figure 5b,c).

## 3. Discussion

HBV genotypes potentially exert a significant influence on clinical outcomes in patients, owing to their correlation with the progression of liver disease [26] and response to antiviral treatment [10,27]. Moreover, infections involving genotype mixtures have been associated with worst clinical outcomes compared to single genotype infections [28]. Notably, NGS techniques allow for the parallel sequencing of thousands or even millions of sequences within a single sample, thereby enabling the detection of genotype mixtures with much greater sensitivity than Sanger sequencing, which yields only a single consensus sequence [29].

In the present study, we utilized a second-generation NGS platform (MiSeq, Illumina) to sequence specific, relatively short regions of the HBV genome. Given the critical importance of selecting optimal genomic regions for accurately discriminating between different genotypes [13], we focused on an amplicon within the preS region of the viral genome for HBV genotype determination, which we had already analyzed by NGS in an earlier study [19]. This decision was based on a previous SW analysis of 400-nt sequences moving forward in 40-nt steps throughout the sequence of a set of 113 whole HBV genome sequences obtained from the GenBank database. This analysis revealed that the preS region showed the highest discriminating capacity for genotyping HBV [15]. Of note, 38.16% of haplotypes obtained from this amplicon displayed gaps in their sequences, mainly due to Dels. The presence of large Del in this region of HBV genome has been previously reported [30,31], and causes gaps in the multiple alignment of haplotype sequences which may result in a loss of their discriminant capacity between different genotypes. This may lead to an ambiguous or incorrect genotypic classification. In contrast, the 5X amplicon did not show any gap and has been successfully employed for HBV genotype determination in previous studies conducted by our group [17,18]. Significantly, our HBV genotyping method relies on utilizing the DB Rule for genotypic classification of each haplotype. Through this distance-based discrimination method, we computed the $\Phi_{t}^{2}$ values of each haplotype to HBV genotypes A–H, representing the genetic distances from each haplotype to each genotype. These genetic distances take into consideration the intraclass variability within each genotype in the sequences of both preS and 5X amplicons. Of note, this variability may be affected by the HBV replicative capacity across different genotypes [32], which may influence the acquisition of mutations. Moreover, it is important to keep in mind that preS and 5X sequences may be subjected to different types of selection pressure (immunological for preS and functional for the 5X), thus potentially conditioning this amplicon-related intraclass variability. The computation of the $\Phi_{t}^{2}$ values of each haplotype to each HBV genotype allowed defining an objective measure of confidence in genotype classification of each haplotype in both amplicons, based on the score $\Phi_{l}^{2}/\Phi_{k}^{2}$ for the 95 reference sequences. Considering the lowest values of these scores, we considered values ≥ 2 as safe for classifying a haplotype into a specific genotype. Remarkably, all haplotypes obtained in preS displayed scores $\Phi_{l}^{2}/\Phi_{k}^{2}>2$, even those with the largest gaps, thereby supporting their reliable classification into their respective genotypes despite the shortening of their sequences. Thus, although the length of the gaps may significantly vary among the viral genotypes, this does not seem to affect the genotypic classification of preS haplotypes.

It is worth noting that Dels causing these gaps affect a region of the P gene that encodes the last aa of the terminal peptide and the N-terminal region of the spacer domain. These regions can accommodate mutations, Ins and large Dels without impacting HBV polymerase function [33,34]. Based on this, the presence of gaps in this portion may have minimal or no impact on viral genome replication. Remarkably, neither preS nor 5X include the region encoding the RT domain, suggesting that antiviral treatment with direct-acting nucleos(t)ide analogs will not have a relevant effect on the genetic variability of their sequences.

Using our NGS-based genotyping method, we genotyped serum or plasma clinical samples from 125 CHB patients, using both preS and 5X amplicons. Many of these patients belonged to immigrant populations with diverse ethnic backgrounds, affording us the opportunity to evaluate our methodology in samples showing viral populations with varied HBV genotypes. In this context, the predominant main genotypes observed in our patient cohort were C and D, both with around 30% of patients, followed by A and E, with less prevalent occurrences of F, B and H. Moreover, three patients displayed potential D/A and D/C recombinants as their main genotypes. We observed that this distribution was related to the HBeAg status of patients, with genotypes A and D significantly associated to HBeAg negativity, and genotype C significantly associated to HBeAg positivity. This different distribution of main genotypes was expected, considering that patients infected with genotypes A and D show earlier and more frequent spontaneous HBeAg seroconversion than those infected with genotype C [35]. Moreover, the main genotypes identified in our patients also corresponded with expectations based on their ethnic backgrounds, according to the patterns of geographical distribution of HBV genotypes [9]. In addition, the distribution of main genotypes in our patient cohort was generally consistent with the genotype distribution observed in a large group of immigrant patients attended at various hospitals across Spain between years 2000 and 2016, recently reported by Aguilera et al. [36], albeit with some disparities. In that study, the prevalent genotypes were D (46.2%), A (21%), E (12.1%) and C (9.3%). Thus, while the most commonly represented genotypes in our patient cohort were consistent with those observed by Aguilera et al., there were variations in the percentages, particularly in genotype C. Notably, Aguilera et al. identified genotype G in less than 1% of patients (not found in our population), with no cases of genotypes I and J or recombinant genotypes. These findings collectively support the reliability of our results and suggest potential differences in the ethnic/geographical origins between the patient populations of Aguilera et al. and ours, as evidenced by variations in genotype percentages between the two cohorts.

In our study, 28/125 samples assessed exhibited mixed genotypes, characterized by minor variants classified into genotypes distinct from the main one identified in preS or 5X. Other studies [16,37] analyzing HBV genome amplicons using NGS have also assessed the proportion of patients showing genotype mixtures. However, comparing results between studies may be difficult due to potential influences from the viral genome regions analyzed [15]. Even if the same region of the viral genome were analyzed, differences in the processing of NGS data could also affect detection of genotype mixtures. In this regard, homogenizing the HBV genotype determination using a sensitive and reliable method, such as the one proposed here, could facilitate the comparison of HBV genotype mixtures observed across different studies.

In this study, all samples showing mixed genotypes, and one sample where no genotype mixtures were detected, exhibited a distinct genotype composition between preS and 5X. The differences observed between genotypes identified in both amplicons suggested the presence of variants originating from intergenotypic recombination in the HBV genome, a phenomenon commonly occurring at different sites (breakpoints), frequently localized to gene boundaries [38]. Since this study focused on two specific HBV genomic regions, it is possible that some potential recombinant variants may have been overlooked. Of note, third-generation sequencing technologies allow for obtaining the whole HBV genome sequence in a single read [39,40], which is an ideal approach for HBV genotyping, especially to identify novel genotypes and intergenotypic recombination [8,12]. In an earlier study [39], four genotype D/E recombination breakpoints were observed in 213 sequences (covering ≥95% of the HBV genome), using the third-generation MinION platform (Oxford Nanopore Technologies). However, even after applying an error correction algorithm to reads passing quality filters, an average error rate of ~12% was obtained.

Another important point of this study was the identification of conserved sequences shared between different viral genotypes. These sequences delineate regions in the viral genome that may play an essential role in the HBV replication cycle or could be valuable in designing novel therapeutic strategies. Following this last point, new antiviral protocols aimed at achieving a functional cure of the infection are currently under development [41]. Some of these protocols involve directly targeting viral transcripts with RNA interference by small-interfering RNAs or anti-sense oligonucleotides, thereby reducing all downstream viral protein and pregenomic RNA production [42]. Therefore, viral replication and the expression of pathogenic proteins, such as the HBV X protein, would be limited. To ensure the success of such treatments, it is important for them to target essential regions in the HBV genome with a high degree of sequence conservation, as a single base mismatch could lead to loss of their effect, as observed in HIV-1 [43]. Keeping this in mind, we utilized data obtained from preS and 5X to identify highly conserved regions in the viral quasispecies across the different genotypes studied. In this analysis, we considered the relative frequencies of haplotypes for calculating the IC. Thus, haplotypes with higher fitness were given greater weight in defining the conserved regions. It is noteworthy that both amplicons were in regions of the viral genome that are the targets of small-interfering RNAs against HBV in clinical trials (the S and *HBX* genes) [42].

For this analysis, we only considered those genotypes that provided sufficient haplotypes for reliably identifying the most conserved windows in their sequences, which limited the study to genotypes A, C, D and E. Despite this, these genotypes collectively account for the majority of hepatitis B infections worldwide: in a meta-analysis of 213 studies covering 125 countries [44], the combined estimated percentages of HBV infections caused by any of these four genotypes accounted for 82.7% of the total. However, the analysis of preS sequence conservation posed challenges due to the presence of gaps with a genotype-specific distribution pattern throughout the entire amplicon sequence. To address this, we narrowed down the analysis to a 144-nt region, limitedly affected by Ins and Del, thus minimizing the occurrence of gaps potentially causing biases when comparing different genotypes. Moreover, given the functional relevance of the aa encoded by this sequence, which constitute the LHBs NTCP-interacting domain [25], it was expected to contain conserved sequence windows. Indeed, we identified 11 highly conserved regions throughout this short preS sequence, but their distribution varied across different genotypes. Notably, we identified three conserved regions in genotypes A (2929–2944), C (2926–2941), and E (2921–2936) that displayed considerable overlap and high sequence identity between them. However, they only partially overlapped with the genotype D conserved region between nt 2881–2894, with only two nt positions present in the regions from all four genotypes. Additionally, a small fraction of genotype A and C haplotypes showed gaps in their respective conserved regions. Taken together, these findings suggest that the region encoding the LHBs NTCP-interacting domain may not be an optimal pan-genotypic antiviral target.

In the 5X sequence, no gaps were detected in any genotype, allowing us to analyze the haplotypes obtained with their full length when studying sequence conservation. Previous studies by our group [17,18] assessed the conservation of the 5X amplicon sequence using a SW analysis of IC with a window length of 25-nt, whereas in the present study we used 12-nt windows. This reduction in window size aimed to achieve greater sensitivity in locating conserved windows common to the four genotypes analyzed. Additionally, here we examined quasispecies sequence conservation in individual genotypes (A, C, D, and E) instead of grouping distinct genotypes together as in our previous studies, thus providing a more accurate determination of the pan-genotypic conserved regions. This analysis revealed two pan-genotypic conserved regions with a high degree of sequence identity. The conserved regions from the different genotypes defining these pan-genotypic conserved regions largely overlapped between positions 1528–1543 in the first region and 1575–1611 in the second. Remarkably, these positions were highly coincident with those identified in our previous analyses (between positions 1519 and 1543, and 1575 and 1603/1605) [17,18]. Conversely, other conserved regions identified in the same studies were not confirmed in all genotypes analyzed in the present study. None of the positions between nts 1255 and 1286 [17,18] were found among the conserved regions in genotype A. Similarly, none of the positions between nts 1545 and 1573 [18], and 1411 and 1435 [17] were found among the conserved regions in genotypes D and C, respectively. Therefore, these results underscore the variability in the degree of conservation in certain regions of the 5X sequence across different genotypes, emphasizing the importance of considering viral genotype when designing RNA interference strategies.

In conclusion, we have developed a method for reliably determining HBV genotypes and identifying their mixtures in clinical samples. This approach is based on the classification of haplotypes obtained by NGS of relatively short regions of the HBV genome, utilizing genetic distance discrimination by the DB Rule. Our NGS-based genotyping method surpasses the Sanger method by enabling the sequencing of each genetic variant present in the viral population and determining their genetic distance to each HBV genotype. This allows for the sensitive detection of possible genotype mixtures and establishes a measure of confidence in genotype discrimination using the score $\Phi_{l}^{2}/\Phi_{k}^{2}$ ratio. Additionally, we observed that sequence conservation may be genotype-dependent, suggesting that genotypes should be individually considered when designing RNA interference strategies. Nevertheless, we identified two highly conserved regions at the *HBX* 5′ end, irrespective of genotype, with high sequence identity, which may be utilized in designing new antiviral RNA interference strategies.

## 4. Materials and Methods

### 4.1. Patients and Samples

All leftover serum or plasma clinical samples (N = 144) obtained from 129 CHB patients were anonymized for subsequent studies. The samples from LCVH included in this study were collected between January and November 2018 from those referred to these clinical laboratories for HBV-DNA levels determination. All samples had HBV–DNA levels ≥ 3 logIU/mL and were utilized solely for the purposes of this project before being discarded. The samples from LCTMS were collected from the immigrant populations attending primary care centers in the southern metropolitan area of Barcelona between 2011 and 2018, all displaying HBV–DNA levels ≥ 3 logIU/mL. The collection of sera was registered in and authorized by the *Instituto de Salud Carlos III* (Ministry of Science and Innovation, Spain) with access number C.0003794.

### 4.2. Serological and Virological Determinations

Serological markers of HBV (hepatitis B surface antigen (HBsAg) and HBeAg) and hepatitis C virus (HCV), HIV and HDV (anti-HCV, HIV and HDV) infections were tested using commercial immunoassays. At the LCVH, the assays to determine HBV and HCV markers were performed on a COBAS 8000 instrument (Roche Diagnostics, Rotkreuz, Switzerland), and anti-HIV antibodies were determined with the Liaison XL murex HIV Ab/Ag kit (DiaSorin, Saluggia, Italy). At the LCTMS, all these markers were determined on a VITROS 5600 instrument (Johnson and Johnson, New Brunswick, NJ, USA). In both centers, anti-HDV antibodies were tested using the HDV Ab kit (Dia.Pro Diagnostics Bioprobes, Sesto San Giovanni, Italy).

HBV-DNA levels were quantified using the Cobas 6800 System with the cobas HBV assay (Roche Diagnostics, Mannheim, Germany) at LCVH and using the Abbott *m*2000 RealTime System with the Abbott RealTime HBV DNA assay (Abbott Laboratories, Des Plaines, IL, USA) at LCTMS.

### 4.3. Amplification and Next-Generation Sequencing of the Hepatitis B Virus Genome

The HBV-DNA was isolated from 250 μL of serum or plasma samples using the automated nucleic acid extraction platform MagNA Pure 24 System (Roche Diagnostics, Mannheim, Germany), following the manufacturer’s protocol.

**Figure 6:**
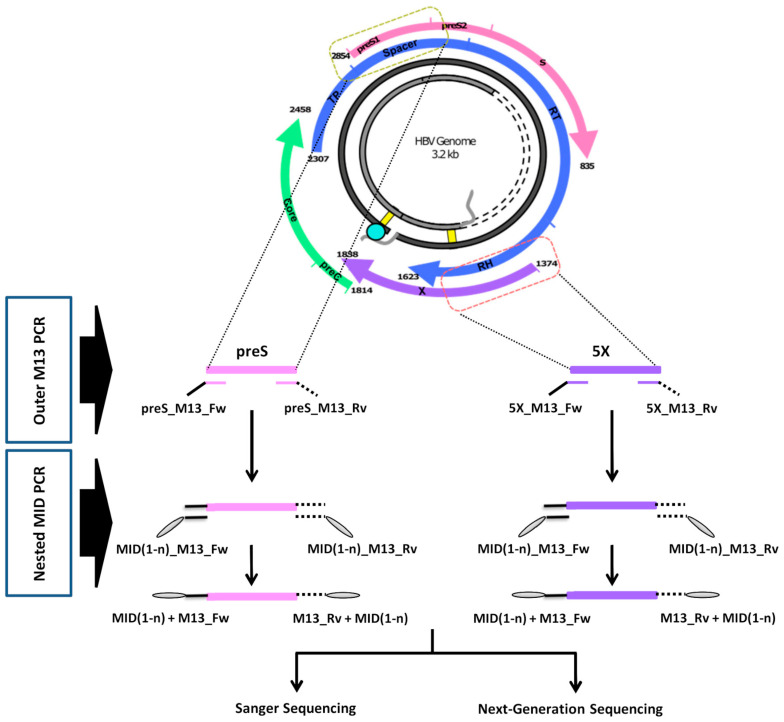
Workflow for amplification of preS and 5X amplicons. The positions in the hepatitis B virus genome of both amplicons are defined by primers preS_M13_Fw and preS_M13_Rv, and 5X_M13_Fw and 5X_M13_Rv, described in the Table 3. The viral genome scheme indicates the regions of the different viral genes overlapped in the sequence of both amplicons: S, depicted in pink, including preS1 and part of preS2 regions in preS; P, shown in blue, including the end of terminal peptide (TP) and most of the spacer coding regions in preS, and most of the RNAse H region (RH) in 5X, which also includes the 5′ end of *HBX* (X), depicted in purple. The final PCR products, including M13 forward (black continuous lines) and reverse (black dashed lines) sequences, and MID(1-n) (grey ovals) at their ends, were all processed with next-generation sequencing. Additionally, a group of 66 samples, with sufficient volume available after next-generation sequencing, were selected for additional Sanger sequencing.

The NGS amplicon libraries were generated in a two-round PCR process using the KAPA HiFi HotStart PCR Kit (KAPA Biosystems–Roche, Cape Town, South Africa), following the workflow depicted in Figure 6. The first round PCR (Outer M13) was performed using the primers preS_M13_Fw and preS_M13_Rv for preS, and 5X_M13_Fw and 5X_M13_Rv for 5X (Table 3). Both preS and 5X PCR mixes underwent the following amplification protocol: initial activation at 95 °C for 5 min; followed by 25 cycles of 98 °C for 20 s, 65 °C for 30 s, and 72 °C for 30 s; and a final extension at 72 °C for 3 min. Subsequently, the PCR products obtained underwent a second round PCR (Nested MID) using the primers MID(1-n)_M13_Fw and MID(1-n)_M13_Rv (Table 3). These primers contained 10-nt sequences at their 5′ ends, referred to as Multiplex Identifiers (MID), which served as unique identifiers in demultiplexing the reads from each amplicon from each patient in each fastq file obtained after NGS. The amplification protocol for this second round PCR comprised an initial activation step at 95 °C for 5 min, followed by 20 cycles of 98 °C for 20 s, 63 °C for 30 s, and 72 °C for 45 s, and a final extension at 72 °C for 7 min.

After the Nested MID PCR, the length of the preS amplicons obtained varied depending on the HBV genotype. The length was 535 nt in genotypes A, B, C, F and H, 502 nt in genotype D, and 532 nt in genotype E (434, 401 and 431 nt, respectively, excluding primer sequences described in Table 3). Regarding the 5X amplicons, their length after the Nested MID PCR was 458 nt (357 nt excluding primer sequences described in Table 3).

The amplification products (preS and 5X amplicons) obtained after the second round PCR underwent purification using magnetic beads (KAPA Pure Beads, KAPA Biosystems–Roche, Cape Town, South Africa), following the manufacturer’s protocol for size selection in NGS Workflows. The purified amplicons were assessed using the Agilent 2200 TapeStation System using the D1000 ScreenTape system (Agilent Technologies, Waldbronn, Germany) and quantified using the Quant-iT Picogreen dsDNA Assay kit (Thermo Fisher Scientific–Life Technologies, Eugene, OR, USA). Both purification and quantification protocols were automated using a Freedom EVO 100 platform (Tecan, Männedorf, Switzerland). The concentration of the 5X and preS amplicons from all samples was normalized to 1 × 10^10^ molecules/µL, with 10 mM Tris-HCl pH 8.0–8.5, and 5 µL (5 × 10^10^ molecules) of each of them were added to different pools. Illumina index adapters were then ligated to the amplicons at both ends in each pool using the KAPA HyperPrep kit (KAPA Biosystems–Roche, Cape Town, South Africa). An additional amplicon library amplification was performed as per the manufacturer’s instructions, along with bead-based cleanup steps (with a beads-to-DNA volumetric ratio of 0.8x) in both size cuts. The amplicons ligated to Illumina index adapters in the pools were quantified by qPCR using the KAPA Library Quantification Kit (KAPA Biosystems–Roche, Cape Town, South Africa) in a LightCycler 480 instrument (Roche Diagnostics, Mannheim, Germany). The concentration of these amplicons was adjusted to 4 nM with PCR-grade water and verified with a second qPCR quantification performed as above. Finally, an appropriate volume of each pool was added to a single final pool. Additionally, the ready-to-use PhiX V3 library (Illumina, San Diego, CA, USA) was diluted to 4 nM and utilized as an internal DNA control. Both the PhiX and the final pool of amplicon libraries were denatured according to manufacturers’ instructions, diluted to 12–15 pM, and combined in a proportion of 80% amplicons final pool and 20% PhiX. This mixture was loaded into a MiSeq Reagent Kit 600 V3 cartridge and sequenced using the MiSeq platform (Illumina, San Diego, CA, USA), following the manufacturer’s instructions.

After sequencing experiments, a fastq file was generated for each Illumina index adapter at each end of the amplicon libraries included in the same pool (R1 and R2). These files have been made openly available in the NCBI Sequence Read Archive (SRA) database, under the BioProject accession number PRJNA913802. The BioSample accession numbers are provided in Table S2.

### 4.4. Sequencing Hepatitis B Virus Amplicons with the Sanger Method

For samples with sufficient available volume (66/144), the amplification products of the preS and 5X Nested MID PCR, as described in the previous section, were also subjected to Sanger sequencing. These amplicons were purified using the ExoSAP-IT PCR Product Cleanup reagent (Thermo Fisher Scientific–Affymetrix, Santa Clara, CA, USA). The sequencing reaction was conducted using the BigDye Terminator v3.1 Cycle Sequencing Kit (Thermo Fisher Scientific–Life Technologies, Austin, TX, USA) with the primers M13_Fw (GTTGTAAAACGACGGCCAGT) and M13_Rv (CACAGGAAACAGCTATGACC), which were positioned at both ends of the Nested MID PCR products (Figure 6), following manufacturers’ instructions. The resulting products were then purified using the BigDye XTerminator Purification Kit (Thermo Fisher Scientific–Life Technologies, Bedford, MA, USA) and loaded onto an ABI PRISM 3130xl Genetic Analyzer (Thermo Fisher Scientific–Life Technologies, Waltham, MA, USA).

As a result, a fasta file was obtained per each primer for the preS and 5X amplicons of each sample. The forward and reverse sequences were aligned, and any mismatches were manually resolved by revising their chromatograms. Finally, both sequences were collapsed into a single consensus sequence for each amplicon in each sample.

### 4.5. Bionformatics Analysis of Sequencing Data

#### 4.5.1. Processing the Next-Generation Sequencing Raw Data

The NGS reads underwent processing using a haplotype-centric data analysis pipeline, which was established in previous studies with HBV and HDV [45], and HCV [46], with some modifications to preserve 5X and preS haplotypes that fully covered the entire amplicons, including those with Ins and/or Del. Essentially, this pipeline comprises the following steps:

1. From fastq files generated by the instrument, full-length 5X and preS amplicons were reconstructed from the corresponding 300-bp paired end reads (R1 and R2) using FLASH [47], imposing a minimum of 20 overlapping bases between them and a maximum of 10% mismatches.
2. Discarding reconstructed reads if 5% or more of their bases had Phred scores below Q30.
3. Demultiplexing reads by matching the sequences of MID(1-n) and 5X or preS forward or reverse specific primers within windows of expected positions. A maximum of one difference in MID sequence and three differences in the specific primer sequences were accepted. Finally, primer sequences were trimmed, and a fasta file was generated for each combination of MID, specific primer, and strand, where identical reads were collapsed to haplotypes with corresponding frequencies derived from the read counts. The reverse haplotypes were reverse complemented.
4. Multiple alignment of forward and reverse haplotypes using MUltiple Sequence Comparison by Log-Expectation (MUSCLE, version 3.8.31, Edgar R.C., Berkeley, CA, USA) software [48], followed by the removal of low-abundance haplotypes (with abundance <0.2% in one or both strands) and those unique to one strand. The common haplotypes were recollapsed and the corresponding coverage was taken as the sum of reads in both strands. These final haplotypes were termed consensus haplotypes and served as the basis of subsequent computations.
5. Minor haplotypes <1% and/or with less than 100 reads were removed. This additional filtering step was applied on the consensus haplotypes to minimize the potential obtention of artifactual low frequency haplotypes in samples.

All computations were conducted in the R language and platform [49], utilizing in-house scripts with the help of the Biostrings [50] and ape packages [51].

#### 4.5.2. HBV Genotype Determination with the Distance-Based Discrimination Method and Selection of Reference Sequences

The genetic distances between amplicon sequences were computed with the Kimura-80 model, a simple model with a single parameter, which distinguishes between transitions and transversions [52]. Based on the distances between pairs of problem sequences and preS or 5X reference sequences for each distinct HBV genotype (A-H), each problem sequence was classified into a genotype by the DB Rule [20]. This method consists in calculating a proximity function ($\Phi_{t}^{2}$) of each problem sequence to each genotype *t*:

(1) $$\Phi_{t}^{2}\left(x\right)=\frac{1}{n_{t}}\sum_{i=1}^{n_{t}}\delta_{i\left(t\right)}^{2}-\frac{1}{2n_{t}^{2}}\;\sum_{i,j=1}^{n_{t}}\delta_{ij\left(t\right)}^{2},\;t=1,\ldots ,g,$$

Each $\Phi_{t}^{2}$ provides the average squared genetic distance of each problem sequence (*x*) to each genotype (*t*), defined by a set of reference sequences *n*_1_…*n_g_* drawn from g genotypes, where:

- $\delta_{i\left(t\right)}^{2}$ is the squared distance from *x* to the *i*-th reference sequence of genotype *t*.
- $\delta_{ij\left(t\right)}^{2}$ is the squared distance between a pair of reference sequences (*i*, *j*) of genotype *t*. The distances between all pairs of reference sequences assigned to a specific genotype were used to compute its geometric variability, which corrects the distance between *x* and the genotype by the intraclass variability of the genotype.

Finally, the assignment of *x* to a genotype was made according to the *k* index giving the lowest $\Phi_{t}^{2}$ (i.e., the genotype to which *x* showed the lowest distance), i.e.:

(2) $${\mathrm{argmin}}_{t}\Phi_{t}^{2}\left(x\right)=\left\{\;k\;|{\;\Phi }_{k}^{2}\left(x\right)\leq \Phi_{t}^{2}\left(x\right),\forall t=1\ldots g\;\right\}$$

The quality of a classification largely depends on the quality of the patterns within each group. Careful attention must be paid in selecting appropriate HBV reference sequences for each genotype. A large set of full-genome HBV sequences with assigned genotypes was downloaded from GenBank. These genomes were trimmed to the positions of preS and 5X amplicons, giving a fasta file for each amplicon. Duplicated sequences, as a side effect from the trimming, were discarded. The sequences in each fasta file were multiple aligned with the MUSCLE software (version 3.8.31, Edgar R.C., Berkeley, CA, USA) [48]. Each sequence was then analyzed to assess its discriminant capacity by classifying it with the DB Rule, using the remaining sequences of the same amplicon. The resulting $\Phi_{t}^{2}$ values for each sequence were sorted and the ratio of two lowest values was computed, $\Phi_{l}^{2}/\Phi_{k}^{2}\geq 1$, providing a classification confidence score. The sequences were then assigned to the genotype with the lowest $\Phi_{t}^{2}$ value, taken here as $\Phi_{k}^{2}$. The reference sequences which resulted in being misclassified (i.e., where the genotype assigned by the DB Rule did not match with the expected genotype as assigned in Genbank) were removed in both amplicons.

#### 4.5.3. Quasispecies Sequence Conservation

The sequence conservation of HBV quasispecies was assessed for the segments of the viral genome covered by the preS and 5X amplicons. This was achieved by computing the information content (IC) of each position in a multiple alignment of all haplotypes classified into genotypes A, C, D and E for each amplicon, as was previously performed by our group [17,18,53]. The IC for each nt sequence position is defined as:

(3) $$IC_{j}=2-\overset{4}{\underset{i=1}{\sum }}p_{ij}\log_{2}\left(p_{ij}\right)$$

IC accounts for the probability of finding a given nt (*i*) at position *j* (*p*_*ij*_) within the set of all four nt (A, C, T and G) taking binary decisions, measured in bits ($\log_{2}\left(4\right)=2$). However, this probability is typically unequal among different nts. For this reason, the uncertainty of *j*; is weighted by the Shannon uncertainty measure or entropy [54]. If Shannon entropy approaches 0 (probability of finding a given nt at *j*; would be close to 1, i.e., highly conserved position), $IC_{J}\approx 2$, whereas if Shannon entropy approaches 2 (all four different nt were almost equally likely to be found, i.e., highly variable position), $IC_{J}\approx 0$.

The most conserved amplicon subsequences, identified in an IC SW analysis, were represented as sequence logos, using the motifStack package in R language [55]. These logos comprise a stack of letters representing each nt identified in each position. The size of the letters indicates the relative frequencies of the nt which they represent at that position, while the height of the stack reflects the IC of that position.

### 4.6. Statistical Methods

All statistical analyses were performed using R Version 4.3.2. [56]. Only *p*-values < 0.05 were considered statistically significant. We employed the two-sample test for equality of proportions without continuity correction to assess the association between the distribution of the main genotypes in relation to the HBeAg status of patients in their baseline samples. The same test was used to compare the proportions of genotype mixtures observed in 5X and preS amplicons.

The statistical differences between different genotypes in the length of gaps of preS haplotypes were assessed using the non-parametric Kruskal–Wallis rank sum test and the post-hoc Dunn test for multiple pairwise comparisons with Bonferroni correction [57].

## Figures and Tables

**Table 1:** Hepatitis B virus genotype determination using the DB Rule in individual haplotypes obtained from preS and 5X amplicons. The table presents the median values of the ratio between the two lowest $\Phi_{t}^{2}$ values ($\Phi_{l}^{2}/\Phi_{k}^{2}$) from each haplotype to specific genotypes, along with the number and percentage of haplotypes classified in each genotype showing gaps.

	preS			5X		
HBV Genotype	N Haplotypes (%) ^†^	Median $\Phi_{l}^{2}/\Phi_{k}^{2}$(IQR)	N Haplotypes with Gaps (%) ^††^	N Haplotypes (%) ^†^	Median $\Phi_{l}^{2}/\Phi_{k}^{2}$(IQR)	N Haplotypes with Gaps
A	147 (14.27)	7.06 (5.17–10.76)	43 (29.25)	226 (27.49)	3.5 (3.13–5.37)	0
B	44 (4.27)	10.33 (9.52–11.09)	2 (4.55)	30 (3.65)	5.5 (5.15–6.24)	0
C	317 (30.78)	7.41 (4.40–12.15)	170 (53.63)	170 (20.68)	6.77 (5.18–10.45)	0
D	405 (39.32)	16.77 (10.94–30.1)	138 (34.07)	236 (28.71)	6.48 (4.76–9.68)	0
E	76 (7.38)	41.34 (18.95–106.89)	40 (52.63)	77 (9.37)	13.94 (6.6–19.68)	0
F	22 (2.13)	4.32 (4.01–4.7)	0	40 (4.87)	6.52 (6.01–7.32)	0
G	−	−	−	−	−	−
H	19 (1.84)	40.47 (30.88 −58.42)	0	43 (5.23)	13.81 (11.18–16.41)	0

**^†^** Percentage for the total preS and 5X haplotypes (1030 and 822, respectively). **^††^** Percentage for the total haplotypes classified within the same genotype, shown in “N haplotypes” column.

**Table 2:** Samples and patients showing genotype mixtures in preS and/or 5X amplicons.

Sample ^†^	preS Main Genotype (% Reads of Sample)	preS Minor Genotypes (% Reads of Sample)	5X Main Genotype (% Reads of Sample)	5X Minor Genotypes (% Reads of Sample)
6	D (100%)	–	A (70.91%)	D (13.96%); C (8.09%); E (7.04%)
7	A (100%)	–	A (66.57%)	C (13.07%); H (11.61%); D (8.75%)
9	F (100%)	–	F (95.77%)	A (4.23%)
11	C (100%)	–	C (91.2%)	A (6.53%); D (2.27%)
12	A (100%)	–	A (92.17%)	C (7.83%)
23	C (97.17%)	F (1.59%); D (1.24%)	C (100%)	–
29	B (96.93%)	D (3.07%)	B (100%)	–
33	B (93.39%)	C (3.50%); A (1.62%); D (1.49%)	B (100%)	–
63	E (98.47%)	D (1.53%)	E (100%)	–
67	D (100%)	–	D (91.40%)	A (5.95%); C (2.66%)
68	F (100%)	–	F (92.51%)	A (7.49%)
70	D (100%)	–	D (90%)	A (10%)
71	D (100%)	–	D (90.30%)	A (9.70%)
74	A (100%)	–	A (97.26%)	D (2.74%)
75	A (100%)	–	A (95.98%)	D (2.53%); C (1.48%)
78	D (100%)	–	D (92.49%)	A (4.53%); E (1.41%); C (1.46%)
82	D (100%)	–	D (66.9%)	A (33.1%)
84	A (100%)	–	A (78.8%)	C (16.55%); D (4.65%)
97	D (98.37%)	C (1.63%)	D (100%)	–
98	D (100%)	–	D (53.64%)	F (46.36%)
102	H (94.35%)	C (5.65%)	H (100%)	–
107	B (100%)	–	B (90.41%)	C (9.59%)
120	E (79.73%)	D (20.27%)	E (100%)	–
121	F (100%)	–	F (97.98%)	C (2.02%)
126	D (97.40%)	C (2.60%)	D (100)	–
131	E (69.18%)	C (30.82%)	E (100%)	–
136	A (78.11%)	C (13.73%); D (8.16%)	A (100%)	–
140	D (100%)	–	A (51.20%)	D (30.11%); C (18.69%)

**^†^** Samples are identified by the numbering in Table S1 for cross-reference.

**Table 3:** Primers used for the obtention of 5X and preS amplicons.

Primer Name	Template Specific Primer Positions	Primer Sequence (5′-3′)
Outer M13 PCR		
preS_M13_Fw	2822–2843 ^†^	GTTGTAAAACGACGGCCAGTGTCACCATATTCTTGGGAACAA
preS_M13_Rv	57–75	CACAGGAAACAGCTATGACCGAACTGGAGCCACCAGCAG
5X_M13_Fw	1234–1254	GTTGTAAAACGACGGCCAGTATGCGTGGAACCTTTGTGGCT
5X_M13_Rv	1631–1612	CACAGGAAACAGCTATGACCATGGGCGTTCACGGTGGTCT
Nested MID PCR		
MID(1-n)_M13_Fw	–	MID(1-n)-GTTGTAAAACGACGGCCAGT
MID(1-n)_M13_Rv	–	MID(1-n)-CACAGGAAACAGCTATGACC

M13, universal adaptor sequence in forward (Fw) and reverse (Rv) primers (underlined sequences). MID(1-n), multiplex identifier sequence, a 10-nt unique identifier for each amplicon from each patient, which allows demultiplexing their reads in a single file. **^†^** Positions in a genotype A genome; in genotypes B, C, D, E and H, positions 2816–2837; in genotype F positions 2815–2836.

## Data Availability

The original data presented in the study (fastq files) are openly available in the NCBI Sequence Read Archive (SRA) database, under the BioProject accession number PRJNA913802.

## Supplementary Materials

The following supporting information can be downloaded at: https://www.mdpi.com/article/10.3390/ijms25105481/s1.
