# Supplementary material for: Genotyping Hepatitis B virus by Next-Generation Sequencing: Detection of Mixed Infections and Analysis of Sequence Conservation
Source: Int J Mol Sci. 2024 May 17;25(10):5481. doi: 10.3390/ijms25105481 (PMC11122360; doi:10.3390/ijms25105481)
Supplement: Supplementary file 1 [file ijms-25-05481-s001.zip › Supplementary Figures.pdf]

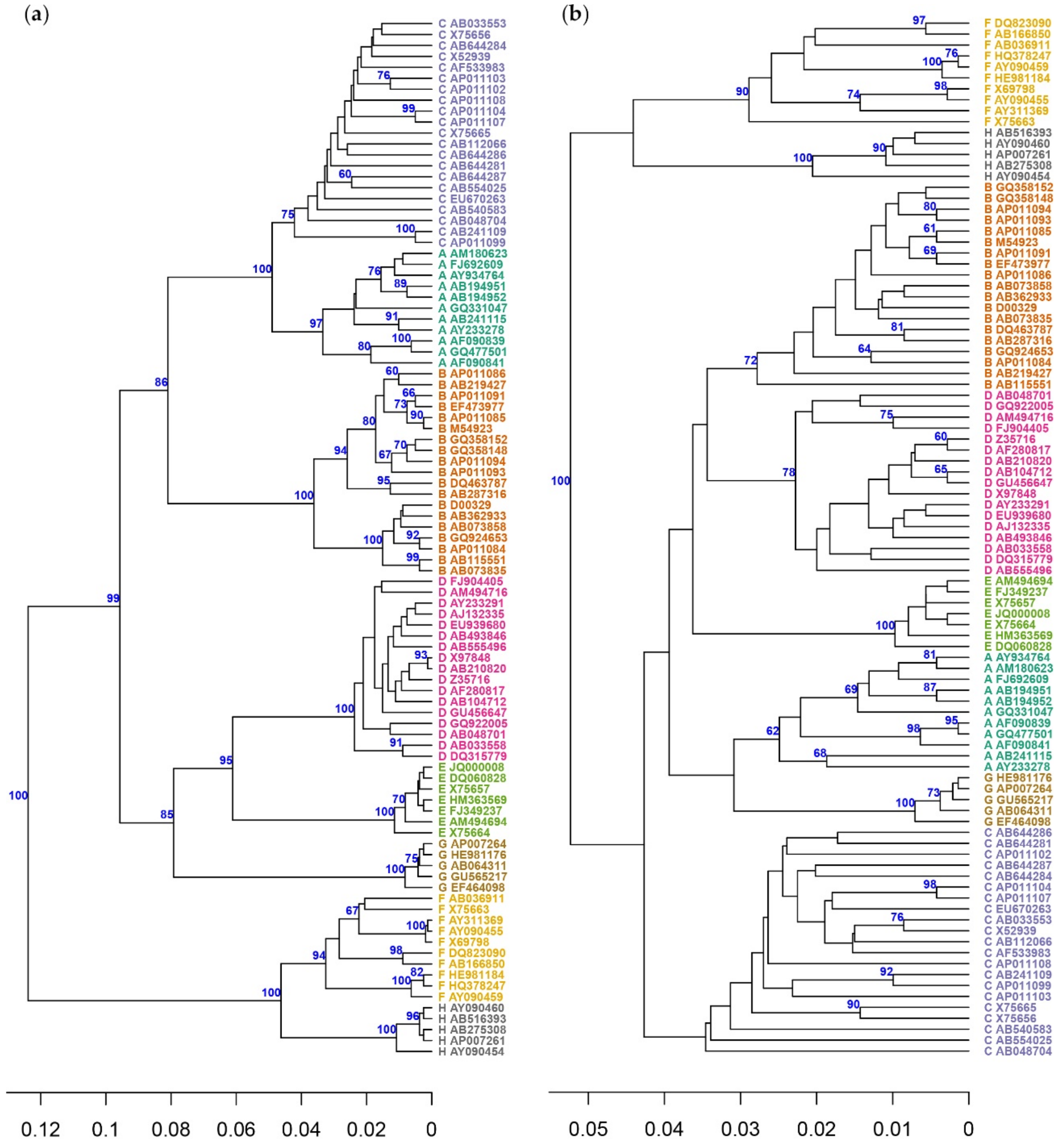

Figure S1. UPGMA phylogenetic trees, based on the Kimura-80 [1] genetic distances, of the two sets of 95 reference sequences (named with their accession numbers) for each amplicon used in the present study: (**a**) preS, covering the entire preS1 and the 5′ end of preS2 coding regions of the hepatitis B virus S gene [nucleotide positions 2837 / 2838 / 2844 / 2874 (genotypes F / B, C, D, E and H / A / G respectively) to position 56]; (**b**) 5X, covering the hepatitis B X gene 5′ end (nucleotide positions 1255 to 1611).

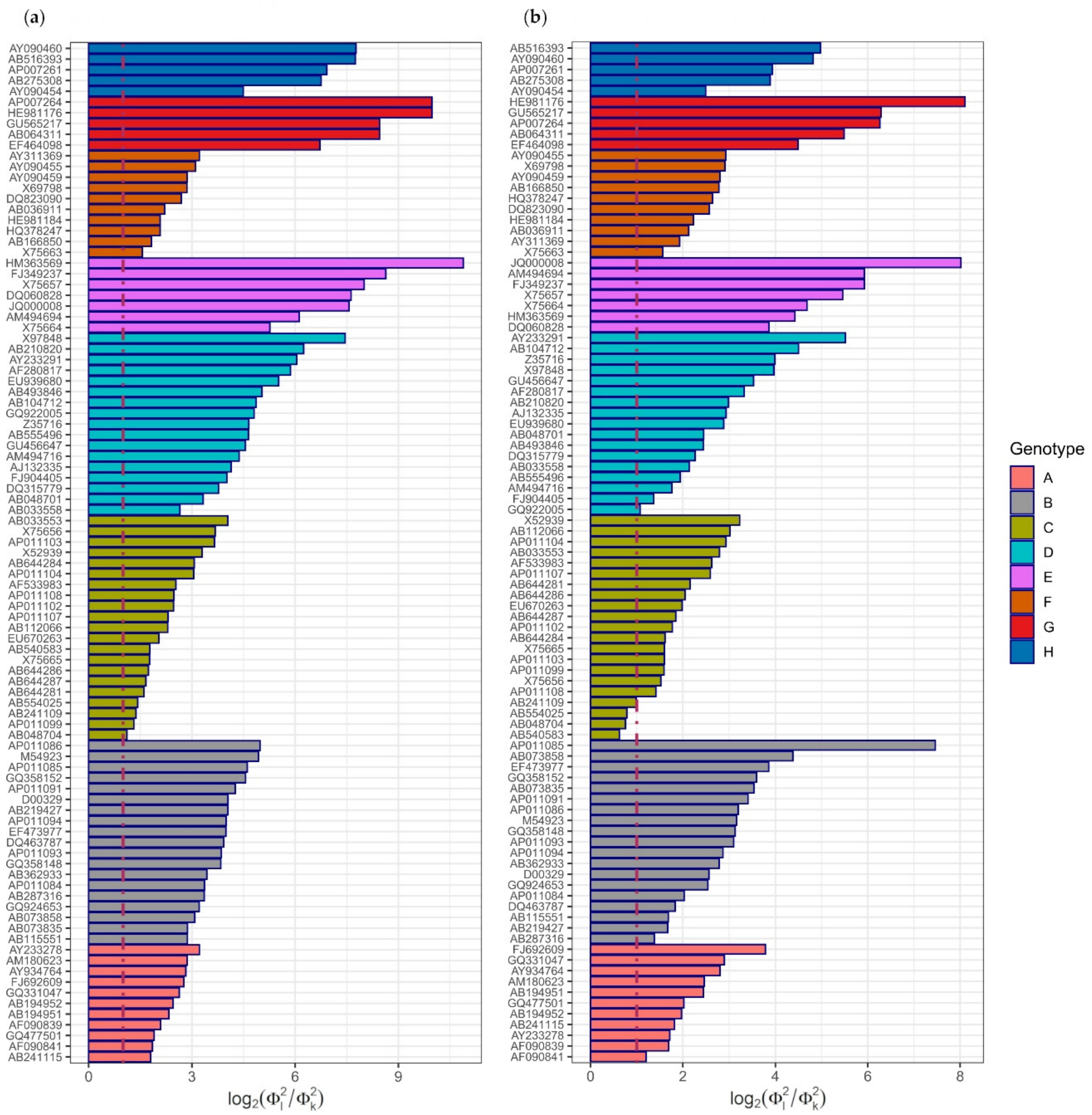

Figure S2. Base-2 logarithm of the ratios between the two lowest proximity function values ($\log_2(\Phi_l^2/\Phi_k^2)$) of each of the 95 reference sequences (named with their accession numbers) representing the hepatitis B virus genotypes A-H, used in the present study, for: (**a**) preS amplicon; (**b**) 5X amplicon; calculated using the Distance-Based discrimination method (DB Rule) [2].

A $\Phi_l^2/\Phi_k^2 \geq 2$ (corresponding to $\log_2(\Phi_l^2/\Phi_k^2) \geq 1$) provided a reliable genotype classification of reference sequences, establishing a classification reliability threshold which was marked as a dashed red line in each plot.

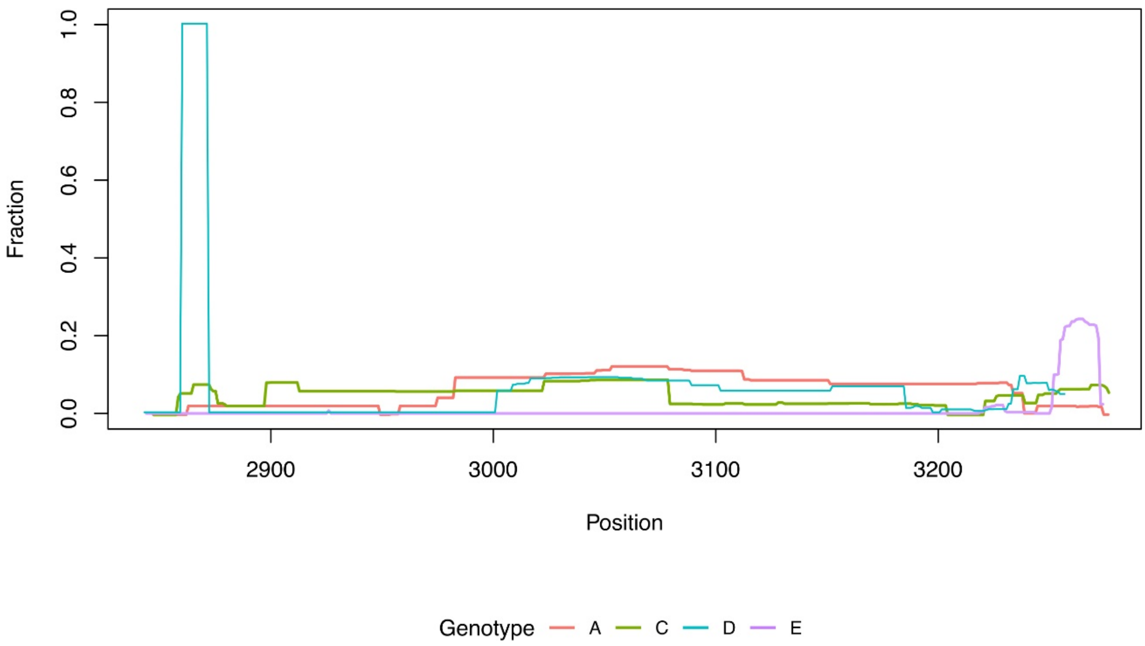

Figure S3. Fraction of gaps by position along the preS amplicon sequence [nucleotide positions 2837 / 2838 / 2844 / 2874 (genotypes F / B, C, D, E and H / A / G respectively) to 56] in a multiple alignment per genotype of haplotypes from genotypes A, C, D and E.

In the multiple alignment of genotype D haplotypes, the presence of a 12-nucleotide insertion in a single haplotype resulted in a gap between positions 2855 and 2866, affecting the remaining haplotypes.

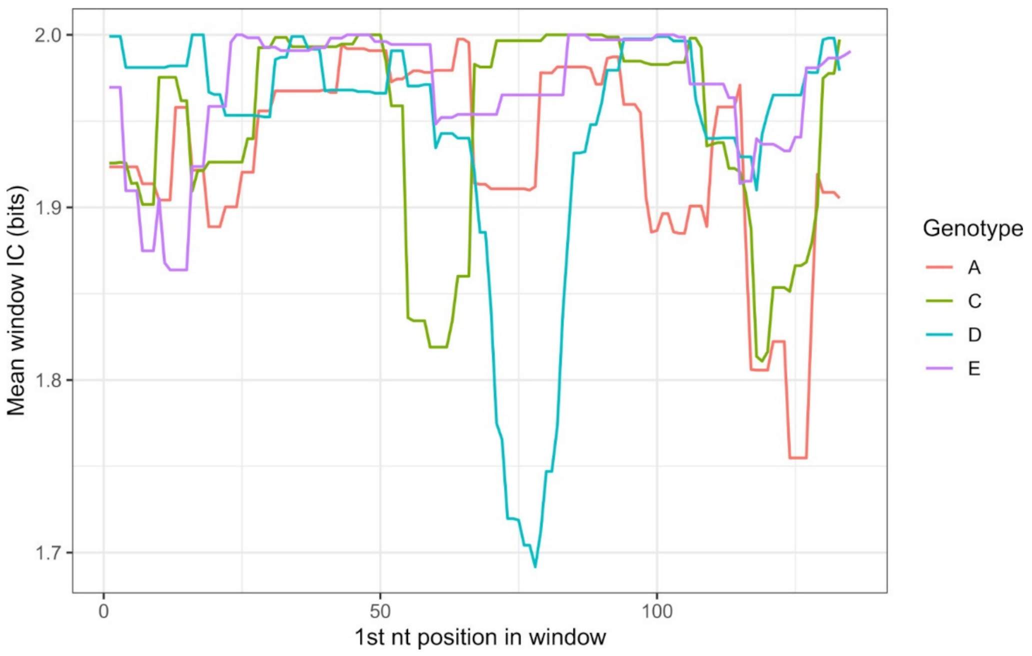

Figure S4. Sliding window analysis of the information content (IC) along the 144-nucleotide (nt) region within the preS amplicon. The analysis covers genotype A, C, D and E haplotypes, encompassing the nt positions: 2887 – 3030, genotype A; 2881 – 3024, genotype C; 2848 – 2991, genotype D; and 2878 – 3021, genotype E. These positions included the sequence encoding the N-terminal domain of the HBV large surface protein responsible for NTCP interaction [3]. The plot illustrates the mean IC values of 133 12-nt windows, moving forward with a 1-nt step between them.

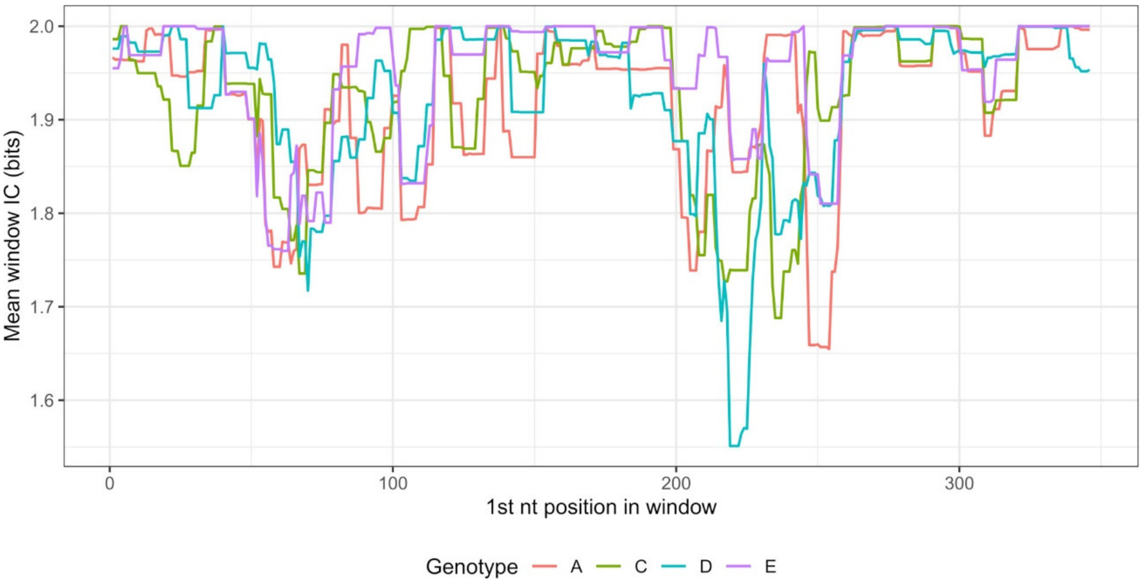

Figure S5. Sliding window analysis of the information content (IC) along the whole 5X amplicon sequence, spanning nucleotide (nt) positions 1255 to 1611, in genotype A, C, D and E haplotypes. The plot displays the mean IC values of 346 12-nt windows, moving forward with a 1-nt step between them.

References

1. Kimura, M. A Simple Method for Estimating Evolutionary Rates of Base Substitutions through Comparative Studies of Nucleotide Sequences. J Mol Evol 1980, 16, 111–120, doi:10.1007/BF01731581/METRICS.

2. Arenas, C.; Cuadras, C.M. Recent Statistical Methods Based on Distances. Contributions to science 2002, 2, 183–191.

3. Yan, H.; Zhong, G.; Xu, G.; He, W.; Jing, Z.; Gao, Z.; Huang, Y.; Qi, Y.; Peng, B.; Wang, H.; et al. Sodium Taurocholate Cotransporting Polypeptide Is a Functional Receptor for Human Hepatitis B and D Virus. Elife 2012, 1, e00049, doi:10.7554/eLife.00049.
